# Content validation and psychometric evaluation of the Angioedema Quality of Life Questionnaire for hereditary angioedema

**DOI:** 10.1186/s41687-023-00576-w

**Published:** 2023-04-03

**Authors:** Magdalena Vanya, Maureen Watt, Saeid Shahraz, Charlotte E. Kosmas, Stephanie Rhoten, Sara Costa-Cabral, Joan Mendivil, Giovanna Devercelli, Karsten Weller

**Affiliations:** 1 ICON plc, South San Francisco, CA USA; 2 Takeda Pharmaceuticals International AG, Zurich, Switzerland; 3 Tufts Medical Center Institute for Clinical Research and Health Policy, Boston, MA USA; 4 ICON plc, Reading, UK; 5 IQVIA, San Francisco, CA USA; 6 Mapi Research Trust, Lyon, France; 7 Takeda Development Center Americas, Inc, Lexington, MA USA; 8 Institute of Allergology, Charité – Universitätsmedizin Berlin, corporate member of Freie, Universität Berlin and Humboldt-Universität zu Berlin, Berlin, Germany; 9 Fraunhofer Institute for Translational Medicine and Pharmacology ITMP, Immunology and Allergology, Berlin, Germany

**Keywords:** Content validity, Hereditary angioedema, Patient-reported outcomes, Validity, Psychometric, Quality of life

## Abstract

**Background:**

There is considerable burden of illness in hereditary angioedema (HAE). However, instruments to assess health-related quality of life (HRQoL) in HAE are limited. The Angioedema Quality of Life Questionnaire (AE-QoL) was developed to measure HRQoL in patients with recurrent angioedema; the validity of the AE-QoL in patients with HAE is described.

**Methods:**

To identify disease-related experiences with a focus on the impact of HAE on HRQoL, interviews were conducted with a group of clinician experts and patients with HAE from Canada, France, Germany, Spain, the United Kingdom, and the United States, along with a targeted literature review. Concepts were mapped to the AE-QoL to assess item relevance, interpretation, and conceptual coverage. Cognitive interviews assessed item clarity and relevance. A psychometric validation was performed using data from a phase 3 trial.

**Results:**

Interviews were conducted with seven clinicians and 40 adult patients. Patients reported 35 unique impacts of HAE on their lives, the most frequent being on work/school, social relationships, physical activities, and emotions, particularly fear/worrying and anxiety. Saturation for these impacts was reached, and all concepts covered in the AE-QoL were reported during the interviews. Patients agreed that the questionnaire items and response options were clear and relevant, and the 4-week recall period was appropriate. The psychometric validation included data from 64 patients. For AE-QoL total scores, excellent internal consistency (Cronbach’s alpha > 0.90), test-retest reliability (intraclass coefficient > 0.80), convergent validity with the Sheehan Disability Scale (r = 0.663), divergent validity with the EQ-5D-5L index (r = 0.292) and EQ-VAS (r = 0.337), and known-groups validity (*p* < 0.0001; ɳ2 = 0.56) were demonstrated.

**Conclusions:**

Qualitative and psychometric analyses showed that the AE-QoL is a reliable and valid instrument for measuring HRQoL in adult patients with HAE from six countries.

**Supplementary Information:**

The online version contains supplementary material available at 10.1186/s41687-023-00576-w.

## Background

Hereditary angioedema (HAE) is a rare genetic disorder with a prevalence of ~ 1:50,000 [1]. Patients with HAE have recurrent, painful swelling of the skin or mucous membranes that may last up to several days. Attacks most commonly affect the face, extremities, and abdomen. Potentially life-threatening attacks that affect the larynx can also occur. Hormonal changes or stressful situations such as surgery may induce attacks; however, many attacks occur without an identifiable trigger [2].

A substantial burden of illness is imposed on patients with HAE, and patients report significant impairment in quality of life [3–7]. Owing to the unpredictable and debilitating nature of attacks, HAE affects daily activities, limits productivity and achievement at work and school, and affects patients’ ability to travel or make and keep plans for future events. The burden of HAE also extends to periods between attacks, as patients report depression and anxiety about experiencing future attacks, the unpredictability of when attacks will occur, and fear of passing HAE on to their children.

Few HAE-specific instruments measure the multidimensional impact of HAE on patients’ lives. Generic instruments that assess health-related quality of life (HRQoL) such as the EuroQol-5 Dimension (EQ-5D) [8, 9] and 12-Item Short Form Survey [10] have not been validated in patients with HAE; thus, their content validity, psychometric properties, and performance in this population are unknown. A number of disease-specific patient-reported outcome (PRO) instruments have been developed and validated for angioedema, including the Angioedema Quality of Life Questionnaire (AE-QoL) [11, 12], the Angioedema Activity Score [13], and the Angioedema Control Test [14, 15]. Instruments developed specifically for the assessment of HRQoL in HAE that have undergone varying degrees of validation include the Hereditary Angioedema Quality of Life Questionnaire (HAE-QoL) [16], the HAE Patient-Reported Outcomes Questionnaire (HAE PRO) [17], and the United States HAE Association QoL Questionnaire (HAEA-QoL [18]); (Supplemental Table S1).

The AE-QoL was the first PRO tool developed and validated to measure HRQoL in patients with recurrent angioedema [11, 12, 19]. It comprises 17 items in four domains (functioning, fatigue/mood, fears/shame, and food) with a 4-week recall period. Although the AE-QoL was validated primarily in patients with chronic spontaneous urticaria, validation also included some patients with HAE. Therefore, it has the potential to be relevant and meaningful among patients with HAE due to the shared symptomatology between HAE and other types of recurrent angioedema [20, 21]. However, further evidence of the content and psychometric validity of AE-QoL in HAE is needed.

This study evaluated the AE-QoL in adult patients with HAE, following the 2009 guidance from the US Food and Drug Administration (FDA) on the use of PROs [22].

## Methods

### Targeted literature review

A targeted literature review was conducted to identify relevant concepts that characterize the patient experience of HAE. Databases were searched to identify peer-reviewed literature published up to 20 July 2016 containing information about patient-reported signs and symptoms of HAE, the impact of HAE on functioning and quality of life, and PROs developed or used for HAE in clinical trials or observational studies (Supplemental Figure S1). Concepts that emerged from the review were then organized into a conceptual model representing the patient experience with HAE, with a focus on impacts related to HRQoL. A follow-up literature review was performed up to 27 April 2020 to capture any PROs developed since the previous search (Supplemental Figure S2).

### Clinician expert interviews

Telephone interviews with clinician experts were conducted by trained qualitative interviewers between March and April 2017 to discuss their patients’ experiences with HAE, including patient-reported symptoms and their impact on patients’ everyday lives. Treatment benefits, factors affecting disease activity, variability in attacks, and recommendations for PRO instruments in HAE were also explored. The interviews followed a semi-structured guide with open-ended questions, and the results were used to develop the patient interview guide.

### Patient interviews

Combined concept elicitation and cognitive interviews were conducted by experienced qualitative researchers (including authors CEK and MV) by telephone between August 2017 and June 2018 with patients with HAE in Canada, France, Germany, Spain, the United Kingdom, and the United States. Patients were recruited by a specialized patient recruitment agency (Global Perspectives, Norwich, UK, and Oviedo, Spain) and were screened before the interview. Patients were identified through the agency’s database as well as through word-of-mouth, internet advertising, email blasts, social media, and patient associations. Interviews lasted up to 90 min and were conducted in the native language of the relevant country. Interviews in Spanish, German, French, and French-Canadian were conducted by interviewers from patient recruitment agencies trained by ICON plc, a Contract Research Organization. Interviews were translated into English and transcribed. Three rounds of interviews were conducted, focusing on the patients’ experience with HAE and its impact on QoL.

Eligible patients had a confirmed diagnosis of HAE type 1 or 2, had experienced an HAE attack in the last 12 months, and were ≥ 9 years old at the time of screening (although the analyses presented include data from adult patients only); (Supplemental Method S1). Patients were excluded if they had HAE with normal C1-inhibitor, or any medical or psychiatric illness or indication of cognitive impairment that could, in the opinion of the study staff, potentially interfere with their ability to participate in the interview. All participant study documents were translated into the patient’s native language using forward translation followed by proof reading and final verification.

Deidentified verbatim transcriptions of the interviews were analyzed using MaxQDA (version 11, VERBI Software, 2016). A codebook was designed to help identify and organize key concepts into common themes. Saturation (the point when no new information is observed in the data) was assessed by developing a saturation matrix of impact concepts reported by patients, which were then mapped onto the AE-QoL to assess item relevance and conceptual coverage.

Cognitive interviews were conducted to evaluate the patients’ understanding of the AE-QoL and to ensure that the content was appropriate for the target population. Linguistically validated licensed translations of the AE-QoL were used (English–United Kingdom, English–United States, English–Canada, French, French–Canada, German, Spanish). Patients completed the questionnaire and were then asked questions regarding the interpretation, clarity, and relevance of each item. Clarity and appropriateness of instructions, recall period, and response options were also assessed. Interview data were analyzed qualitatively.

### Quantitative validation

A psychometric validation was conducted using data from a phase 3 clinical trial that evaluated the efficacy and safety of subcutaneously administered C1-inhibitor for the prevention of HAE attacks in adults and adolescents (NCT02584959) [23]. The study used a three-period, three-sequence crossover design (A/B, B/A, A/A) where treatment A was 2000 IU C1-inhibitor administered twice weekly and treatment B was placebo. The duration of each period was 14 weeks. For each period, patients completed the AE-QoL and Sheehan Disability Scale (SDS) [24] at baseline (Day 1 before treatment administration) and every 4 weeks (Weeks 5, 9, and 13), and the EQ-5D 5-level descriptive system (EQ-5D-5L) including the EQ visual analog scale (EQ-VAS) [8] at baseline and Week 12. Pooled data for adult patients from the first period of the study were used for the psychometric validation analyses.

Internal consistency of the total score and each domain score was measured to evaluate the homogeneity of the items within the AE-QoL. Scores at baseline were assessed using Cronbach’s alpha with a minimum acceptable alpha of 0.70 [25]. Item-total correlations (the relationship between individual item scores and the total score) were assessed using Spearman’s rank correlations; 0.30 [26] was the minimum correlation, and a strong correlation was predefined as ≥ 0.50.

Test-retest reliability was assessed for the total and domain scores at Weeks 9 and 13, as patients were assumed to be stable at these time points; a paired t-test was used to examine any significant differences in scores. A two-way, mixed-effects intraclass coefficient (ICC) was used to test the degree of correlation for the assessment of test-retest reliability, with thresholds ≥ 0.75 interpreted as good to excellent [25].

Convergent and divergent validity were examined at baseline using Spearman’s rank correlations to assess the extent to which baseline AE-QoL scores were associated with the SDS, EQ-5D-5L, and EQ-VAS. Correlation coefficients ranging from 0.10–0.29, 0.30–0.49, and 0.50–1.0 were classed as weak, moderate, and strong correlations, respectively [27, 28].

Known-groups validity was assessed by comparing mean AE-QoL total and domain scores at Week 5 between tertile subgroups of increasing severity, as defined using SDS. Differences between subgroups were examined using analysis of variance (ANOVA). Effect sizes (eta squared, ɳ2) were calculated to establish the magnitude of the difference between subgroups (i.e., ɳ2 = sum of squares between groups/total sum of squares). An ɳ2 of 0.01–0.05, 0.06–0.13, and ≥ 0.14 was considered a small, moderate, and large effect, respectively [27].

Sensitivity to change from baseline to Week 5 and Week 13 was evaluated using Spearman’s rank correlations between change in AE-QoL and SDS scores. Correlation coefficients were classified in a way similar to the convergent and divergent validity analyses. Meaningful change (the smallest amount of change on the AE-QoL likely to be important based on the statistical characteristics of the sample) was explored using the distribution of AE-QoL scores at Week 5. Week 5 data were used because of the smaller sample size compared to baseline, thus providing a more conservative estimate of the distribution-based parameters. Three criteria were applied: 0.5 standard deviation (SD) [29], standard error of mean (SEM), and minimal detectable change (MDC). SEM and MDC were calculated using results from internal consistency (alpha) and test-retest reliability (ICC thresholds).

Data analyses were performed using SAS Version 9.4 (SAS Institute Inc., Cary, NC, USA).

## Results

### Targeted literature review

The literature search initially identified 28 relevant publications for full-text review (Supplemental Figure S1). Numerous symptoms and impacts associated with HAE were identified, showing that the patient experience varies greatly between patients (Supplemental Tables S2–S4). Of 79 HAE-related impacts identified, 31 were associated with HAE attacks, 33 were experienced between attacks, and 15 were treatment-related. The most frequent attack-related impacts were missing work (35.7%) and impaired ability to perform or complete daily activities (14.3%); the most frequent between-attack impacts were hindered advancement at work/school (21.4%), depression (21.4%), fear/worrying (17.9%), and anxiety (17.9%). Hospitalization (28.6%) along with side effects (17.9%), unnecessary treatments or surgical procedures (17.9%), and a need for emergency department visits (17.9%) were the most frequent treatment-related impacts.

The literature search identified four disease-specific PRO measures developed to evaluate HRQoL in HAE: the AE-QoL, HAE-QoL, HAE PRO, and the HAEA-QoL. Among the 31 publications extracted for full review in the updated literature search (Supplemental Figure S2), the AE-QoL was the most frequently reported PRO instrument, including use in six clinical trials [30–35].

### Clinician expert interviews

Interviews were conducted with seven clinical experts based in Canada, Germany, Spain, and the United States. Five experts specialized in allergy and immunology, and two specialized in dermatology and allergy medicine. They had a mean (range) of 16 (10–20) years of experience treating patients with HAE and had seen/treated a mean (range) of 39 (12–89) patients with HAE in the past 12 months.

The experts reported 16 different signs and symptoms associated with HAE attacks, including swelling and pain, difficulty breathing, and nausea/vomiting (Table 1). All experts stated that their patients with HAE experienced anxiety/fear/depression and impacts on work and school as a result of attacks. Four experts stated that they used disease-specific PRO instruments to assess the health of their patients with HAE and that they were using, or had used, the AE-QoL. The length of recall and short time to completion were important factors in deciding which instrument to use, and specificity for angioedema was mentioned by some experts as a key reason for using the AE-QoL.

**Table 1:** Signs/symptoms and HRQoL impacts on patients, as reported by clinical experts

Sign/symptom or impact	*N* = 7
HAE signs/symptoms^a^	
Swelling	7
Pain (general, abdominal, facial)	7
Difficulty breathing/suffocating	6
Nausea/vomiting	6
Bloating	4
Rash/erythema	4
Swallowing difficulties	3
Voice change/dysphonia	3
Talking difficulties	2
Skin tightness	2
HRQoL impacts^b^	
Anxiety/fear/depression	7
Work/school	7
Concern for family history (e.g., passing on HAE to children)	6
Mobility/functioning	6
Leisure/daily activities	6
Travel/vacation	5
Psychological issues	4
Social life/relationships	4
Food triggers/loss of appetite	2
Tiredness/fatigue	2

*HAE* hereditary angioedema; *HRQoL* health-related quality of life

^a^ The following signs/symptoms were each reported by one clinical expert: headache, diarrhea, cramping, skin irritation, bowel wall obstruction, numbness/tingling

^b^ The following HRQoL impacts were each reported by one clinical expert: sleep disorder, swallowing, stress, problems with urination/bowels, concentration problems

### Patient interviews

Forty adult patients with HAE (88% female, 90% White; mean [range] age of 39 [18–66] years) were interviewed. They reported an attack frequency ranging from 3 to ≥ 100 attacks per year (Supplemental Table S5). The most common symptoms of attacks were nausea/vomiting (n = 30), pain (n = 27), and swelling (n = 27) (Supplemental Table S6). Of 13 patients who were asked about how they felt between HAE attacks, more than half (n = 8; 61.5%) reported feeling normal/good; for example, one patient reported “*When I don’t have an attack, I just feel completely normal. Like, you could completely forget that I even had HAE and I wouldn’t notice any difference […]” (United Kingdom).* Tiredness/fatigue and negative feelings or emotions such as irritability and anger or frustration were each mentioned by three (23.1%) patients. For example, one patient described “*[…] a lot of tiredness, very, very emotional. You can go snappy as well, where you’re very anxious. You feel quite imbalanced, to be honest like, not crazy, but you feel – it’s like you’re constantly waiting for that attack, if it’s going to happen. Am I going to get an attack today? You know, is this going to give me an attack? You’re, kind of, living on edge, so, you’re quite highly strung and I find a lot of my family, and other people I know with the condition, we all seem to be quite similar like that” (United Kingdom)*.

Patients reported 35 unique impacts of HAE on different aspects of their lives (Table 2). At least half of patients described impacts on work (58%), social relationships (55%), and physical exercise (50%). Emotional impacts were also frequently described, including stress/worry (43%), fear/anxiety (33%*)*, and shame/embarrassment (33%). In addition, patients reported that they limited their participation in desired activities in the hope of preventing another attack, and felt bad over missing events or activities because of an attack. Saturation for the 35 impacts was reached by the 38th interview (N = 40); 83% and 91% of impacts were mentioned by the 5th and 14th interviews, respectively.

**Table 2:** Impacts of HAE reported by patients during interviews

Impact, *n* (%)	Patients (*N* = 40)	Sample patient interview quotes
Basic physical functions		*“[…] when my foot is very swollen, I can’t walk anymore.” (France)*
Walking	19 (48)	
Use of hands or arms	9 (23)	
Standing	5 (13)	
Sitting	3 (8)	
Activities of daily living		*“They were abdominal attacks and they put me down in bed. I can’t get up […]”(United States)*
Personal hygiene and dressing	9 (23)	
Bathing and showering	4 (10)	
Getting out of bed	4 (10)	
Getting to or using the toilet	2 (5)	
Instrumental activities of daily living		*“[…] if there is an attack on my feet, for example, I cannot drive […] I could have gone to work but I could not move, I could not walk, I could not drive […] I had to take sick leave.” (France)*
Driving	8 (20)	
Cleaning the house or doing laundry	7 (18)	
Shopping	4 (10)	
Cooking	4 (10)	
General household activities	1 (3)	
Emotional impact		*“[…] since the time my face swelled up, it seems as though I’ve developed a kind of fear or anxiety that it might happen again, because in the face it’s most scary, because it’s so painful when the mouth and lips swell up and the fear is that it might go down into the airways and you won’t get to the hospital in time. So you develop a kind of anxiety, phobia, that it might come back […]” (Canada)*
Stress or worry	17 (43)	
Fear and anxiety	13 (33)	
Shame or embarrassment	13 (33)	
Frustration	6 (15)	
Depression/sadness	5 (13)	
Hereditary concerns	4 (10)	
Anger	3 (8)	
Irritability	2 (5)	
Work/school		“*[…] I am exhausted by my attacks and I realized that it’s no longer easy to work, in the end.” (France)*
Work	23 (58)	
School or university	9 (23)	
Social		*“[…] Well, there are moments where I completely isolate myself…I don’t leave the house, and I don’t see anybody and I certainly don’t want to see anybody. But even between the attacks, sometimes, there are times when I’m really tired, so I’m not in the mood to go out, I’m not in the mood to see anybody […].”(France)*
Relationships	22 (55)	
Sleep and energy levels		*“[…] I have a lot of pain before the attack, I already feel it in my body. I have a lot of joint pain. I’m tired, I sleep a lot.” (Canada)*
Low energy levels or tiredness	9 (23)	
Difficulty sleeping	5 (13)	
Sleeping too much	2 (5)	
Recreation		*“I also can’t do exercise if I’ve had an attack […] I love to do yoga, but I can’t do that if I’ve had an attack ‘cause it just puts too much pressure on points […] if you’ve had like an arm swelling, you can’t put that down on the ground and support yourself […].” (United Kingdom)*
Physical exercise	20 (50)	
Leisure activities	13 (33)	
Ability to travel	12 (30)	
Eating and drinking		*“I now know that tomatoes is a problem […] the amount of time I spend telling people in restaurants I’m allergic, answering weird questions about it from my friends and/or family, sending things back because they’ve brought me something with tomatoes…[the] inconvenience and worry and anxiety and just like, one more thing to think about and that extra thing that’s annoying.” (United Kingdom)*
Eating and drinking difficulties	11 (28)	
Dietary restrictions	7 (18)	
Managing/living with HAE		*“[…] I never really RSVP’d to anything because I never knew when the next bout would occur, or I had to cancel on short notice […].” (Germany)*
Ability to plan ahead	15 (38)	
Difficulties related to clothing	9 (23)	
Lack of concentration	1 (3)	

*HAE* hereditary angioedema

All concepts covered by items in the AE-QoL were reported by patients during the interviews. The AE-QoL domains captured the most prevalent and important impacts in this study population, except for travel. An effect on ability to travel was described by 30% of patients; however, the travel-related issues generally pertained to long-distance travel, which is not a common event for most individuals. Issues included travel disruption due to an attack, and challenges in planning, such as managing medication and ensuring the adequate availability of medical care at the destination.

Overall, the majority of patients understood and interpreted the AE-QoL instructions, items, response options, and recall period as intended and without any problems. Patients noted that the instructions are *“pretty simple…and pretty self-explanatory” (United States)* and *“pretty easy to understand” (United States)*. On average, 93% of patients (78–100% across specific items) felt that every item on the AE-QoL was straightforward and easy to interpret, and 86% (71–100% across specific items) thought that every item was relevant to their experience with HAE. Most patients considered the 4-week recall period appropriate given the episodic nature of HAE and the type of questions asked.

### Psychometric validation

Data from 64 adult patients (69% female, 92% White; mean [range] age 41 [13–72] years) were included in the psychometric validation analyses. The mean (SD) baseline AE-QoL total score was 46.60 (20.89), indicating a moderate-to-large effect of HAE on HRQoL [19]. The baseline total and domain scores showed high variability in HRQoL impairment among the patients (Supplemental Figure S3).

Excellent internal consistency was found for the total score (Cronbach’s alpha = 0.92), and alpha ranged from 0.78 to 0.88 for the domain scores. Alpha was > 0.90 for the total score with each item deleted and was > 0.75 for domain scores with each item deleted (Table 3). Item-total correlations showed a strong correlation for the total score (r ≥ 0.52) and for domains (r ≥ 0.56) (Supplemental Table S7).

**Table 3:** Summary of Cronbach’s alpha with each item deleted for AE-QoL total and domain scores at baseline

AE-QoL item (*n* = 64)	Cronbach’s alpha with each item deleted (raw^a^)				
	Total score	Functioning	Fatigue/Mood	Fears/Shame	Food^b^
Item 1 (Impairment of work)	0.91	0.87	-	-	-
Item 2 (Impairment of physical activity)	0.92	0.82	-	-	-
Item 3 (Impairment of spare time activities)	0.91	0.81	-	-	-
Item 4 (Impairment of social relations)	0.92	0.83	-	-	-
Item 5 (General limitations in foods and eating)	0.92	-	-	-	N/A
Item 6 (Difficulties of falling asleep)	0.92	-	0.80	-	-
Item 7 (Waking up during the night)	0.92	-	0.79	-	-
Item 8 (Feeling tired during the day)	0.91	-	0.76	-	-
Item 9 (Difficulties in concentrating)	0.92	-	0.81	-	-
Item 10 (Feeling depressed)	0.92	-	0.83	-	-
Item 11 (Limitations in the selection of food and beverages)	0.92	-	-	-	N/A
Item 12 (Feeling burdened at having swellings)	0.91	-	-	0.86	-
Item 13 (Fear of new suddenly appearing swellings)	0.91	-	-	0.85	-
Item 14 (Fear of increased frequency of swellings)	0.91	-	-	0.84	-
Item 15 (Ashamed to visit public places)	0.91	-	-	0.84	-
Item 16 (Embarrassed by the appearance of swellings)	0.92	-	-	0.85	-
Item 17 (Fear of long-term negative drug effects)	0.92	-	-	0.89	-
Cronbach’s alpha	0.92	0.87	0.83	0.88	0.78

*AE-QoL* Angioedema Quality of Life Questionnaire

^a^ All standardized Cronbach’s alpha estimates were consistent with the raw Cronbach’s alpha estimates (i.e., within 0.01)

^b^ Cronbach’s alpha with each item deleted not calculated for domains with ≤ 2 items

For test-retest reliability, the mean 4-week change in the AE-QoL total and domain scores was minimal; the largest change was in the food domain (mean [SD]: −2.98 [20.99]) (Table 4). No significant differences were found between scores over the 4 weeks (*p* > 0.05 for all domains). ICC values for the total and domain scores were all > 0.80 with upper limit of 95% CI ≥ 0.90, indicating good to excellent test-retest reliability.

**Table 4:** Test-retest reliability of AE-QoL total and domain scores at Week 9 and Week 13

AE-QoL score (*n* = 42)	Week 9 mean (SD)	Week 13 mean (SD)	Mean change (SD)	t-test statistic, *p* value	ICC (95% CI)
Total score	33.37 (23.33)	33.02 (21.83)	−0.35 (14.37)	−0.16, 0.8753	0.89 (0.80–0.94)
Functioning	25.00 (25.94)	26.34 (28.84)	1.34 (21.23)	0.41, 0.6848	0.83 (0.68–0.91)
Fatigue/Mood	32.74 (23.01)	30.00 (21.21)	−2.74 (17.71)	−1.00, 0.3222	0.81 (0.65–0.90)
Fears/Shame	39.68 (29.78)	41.07 (28.66)	1.39 (19.25)	0.47, 0.6426	0.88 (0.78–0.94)
Food	32.74 (31.59)	29.76 (32.78)	−2.98 (20.99)	−0.92, 0.3635	0.88 (0.78–0.94)

*AE-QoL* Angioedema Quality of Life Questionnaire; *ICC* intraclass coefficient

All relationships between AE-QoL and SDS scores were moderately to strongly associated (*p* < 0.001), demonstrating convergent validity. The largest associations were found between SDS total scores and AE-QoL total scores (r = 0.663) (Table 5). Correlations between the AE-QoL total score and EQ-5D-5L index and EQ-VAS scores were 0.292 and 0.337, respectively, indicating weak to moderate correlation and supporting divergent validity. Results for AE-QoL domain scores were similarly weak to moderate.

**Table 5:** Convergent and divergent validity correlations^a^ at baseline between the AE-QoL and SDS, EQ-5D-5L, and EQ-VAS

Measure	*N*	AE-QoL total	Functioning	Fatigue/Mood	Fears/Shame	Food
SDS						
Total	62	0.663***	0.612***	0.507***	0.521***	0.504***
Work/school	62	0.581***	0.548***	0.466***	0.446***	0.425***
Social life/leisure activities	64	0.645***	0.569***	0.456***	0.561***	0.534***
Family life/home responsibilities	64	0.674***	0.621***	0.546***	0.524***	0.495***
EQ-5D-5L						
Index	64	0.292*	0.183	0.368**	0.227	0.167
VAS	64	0.337**	0.147	0.328**	0.373**	0.187

*AE-QoL* Angioedema Quality of Life Questionnaire, *EQ-5D-5L* EuroQol 5-Dimensional 5-Level Descriptive System, *SDS*, Sheehan Disability Scale, *EQ-VAS* visual analog scale

*p < 0.05, **p < 0.01, ***p < 0.001

^a^ Absolute Spearman’s rank correlations

Known-groups validity was supported with significant differences observed between the tertile subgroups concerning AE-QoL total and domain scores (*p* < 0.001) (Table 6). Effect sizes were large for the total score (ɳ2 = 0.56) and the domains (ɳ2 range: 0.29–0.63). In addition, a linear trend was observed between the tertile subgroups, as mean AE-QoL total and domain scores increased with higher SDS severity.

**Table 6:** Differences in AE-QoL scores at Week 5 between ranked SDS tertile groups of increasing severity for known-groups validity

SDS Total (*n* = 45)	Mean (SD)				
	AE-QoL total	Functioning	Fatigue/Mood	Fears/Shame	Food
Tertile group					
Tertile 1 (*n* = 15)	19.02 (15.51)	5.83 (11.20)	25.67 (25.35)	23.89 (19.89)	14.17 (17.59)
Tertile 2 (*n* = 16)	32.44 (17.17)	26.17 (18.43)	32.50 (19.49)	37.50 (22.00)	29.69 (21.35)
Tertile 3 (*n* = 14)	63.13 (17.32)	59.38 (20.62)	59.29 (24.33)	70.83 (22.35)	57.14 (30.51)
ANOVA *p* value	< 0.0001	< 0.0001	0.0008	< 0.0001	< 0.0001
ANOVA *p* value (linear trend)	< 0.0001	< 0.0001	0.0003	< 0.0001	< 0.0001
Eta squared,^a^ ɳ2	0.56	0.63	0.29	0.47	0.37

*AE-QoL* Angioedema Quality of Life Questionnaire, *ANOVA* analysis of variance, *SDS* Sheehan Disability Scale

^a^ ɳ2 equal to 0.01 to 0.05 considered a small effect, 0.06 to 0.13 a moderate effect, and 0.14 and over, a large effect [27]

Longitudinal analysis using two time points also provided support for the ability of the AE-QoL to detect change, with moderate to strong correlations between changes in AE-QoL and SDS total scores from baseline to Week 13 (r = 0.40, *p* < 0.05), as well as Week 5 to Week 13 (r = 0.51, *p* < 0.01). In addition, ability to detect change was generally supported for the domain scores (r range: 0.35–0.42, all *p* < 0.05), except for lower correlations for fatigue/mood (*r* = 0.23, *p* > 0.05) and fear/shame domain scores (r = 0.30, *p* > 0.05) using the change between baseline and Week 13 (Supplemental Table S9). Distribution-based estimates of meaningful change for the AE-QoL total score ranged from 5.35 to 18.70, with 0.5 SD = 11.87 and SEM = 5.35 (both were estimated at Week 5). These are larger than previously proposed estimates when considering the variability estimates based on Cronbach’s alpha (Supplemental Table S9).[12].

## Discussion

Although several generic instruments that measure various aspects of HRQoL have been widely used, disease-specific instruments are preferable because they are more sensitive to change, covering disease-specific concepts relevant to the specific population. Based on findings from the literature review and clinical expert interviews, the AE-QoL was identified as the most appropriate tool for clinical studies of patients with HAE and is also the most frequently used tool in HAE clinical trials. Initial development and validation of the AE-QoL instrument [11] included a small number of patients with HAE types 1 and 2 (as expected, given that HAE is a rare disease), and concept saturation was not documented. Furthermore, it took place at two specialized centers for angioedema in Germany, so potential geographical or cultural differences were not captured. This current study sought to establish further content validity and measurement properties for the AE-QoL in an expanded HAE adult patient population.

Findings from the literature review and from interviews with clinicians and patients underscored the significant disease burden experienced daily by patients with HAE. The most frequent individual HRQoL impacts were on work, social relationships, and physical exercise, along with emotional impacts such as stress/worry, fear/anxiety, and shame/embarrassment. Concept saturation was achieved during the patient interviews and elicited concepts mapped closely to the AE-QoL item content. Overall, most patients understood and interpreted items in the questionnaire as intended. Together, these results support the relevance, comprehensiveness, and content validity of the AE-QoL for assessing HRQoL in adult patients with HAE.

The quantitative analysis showed the AE-QoL to be a reliable measure with acceptable levels of internal consistency and test-retest reliability. The AE-QoL also demonstrated strong convergent validity with the SDS and divergent validity with the EQ-5D-5L index and EQ-VAS, as well as known-groups validity. In addition, sensitivity to change was demonstrated using longitudinal analysis with two time points. Of note is the difference in the recall period between the AE-QoL (4 weeks) and the other instruments (SDS, 1 week; EQ-5D-5L, 24 h), which may have contributed to the level of correlations observed, particularly for the sensitivity to change analysis, and may also explain the divergence with the EQ-5D-5L. These results provide strong evidence for the psychometric properties of the AE-QoL, further confirming the content validity, reliability, construct validity, and responsiveness of the AE-QoL in patients with HAE [11].

The burden of illness and the impact of HAE on HRQoL have recently become more recognized and understood [3–7]. Given the extensive heterogeneity in HAE and the unpredictability of attacks, an individualized approach to management would be beneficial. Furthermore, therapeutic strategies should not only target the treatment of attacks but should also aim to minimize the effect on HRQoL experienced between and during attacks, as demonstrated by findings from patient interviews in this study and other published patient testimonies [36–39]. This underlines the importance of measuring HRQoL as well as other patient-centric endpoints in HAE to demonstrate meaningful treatment benefit from the patient’s perspective; this is mandated by the 21st Century Cures Act [40] and further operationalized in the FDA’s guidance on Patient-Focused Drug Development [41–44], elaborating on the principles proposed in the FDA PRO Guidance [22]. Accordingly, well-established content validity and robust measurement properties, including sensitivity and the ability to detect change, are critical evidence of the PRO tool being suitable for measuring treatment benefit in the target population [22, 45].

Limitations of this study include the fact that the psychometric analysis was retrospective and the sample size was small; the small size is a common disadvantage of studies in rare diseases. The patient population was predominantly female; while this is consistent with numerous clinical trials [32–35, 46] and other studies [3, 47, 48] in which a higher number of female patients with HAE was enrolled or reported, the disease experience in females may differ from that in male patients [49]. Although sensitivity to change and meaningful change for the AE-QoL were established previously [12], anchor-based analyses of meaningful change thresholds were limited in this study, as there were no global assessments of symptom severity or change to use as anchors. Distribution-based methods performed here are to be used only as a complement to anchor-based methods. For example, a minimal clinically important difference in AE-QoL total score of 10.5 was previously reported based on the 0.5 SD calculation [12], compared with 11.87 in this analysis. Thus, although conclusions on the clinical relevance of the findings here rely on distribution-based results, they support previous responder estimates and may be useful for supporting future analyses to define responders. Finally, the results described herein were based on data from adult patients with HAE; a future analysis of content validity and psychometric properties of the AE-QoL in pediatric patients would be beneficial.

## Conclusions

Using both qualitative and quantitative methods, the AE-QoL was demonstrated to be an appropriate, interpretable, and reliable tool for assessing HRQoL in a cross-cultural population of adult patients with HAE.

## Electronic supplementary material

Below is the link to the electronic supplementary material.

**Additional file 1:** Supplemental Tables S1–S9. Supplemental Figures S1–S3.

## Data Availability

The datasets, including individual participant data supporting the results reported in this article, will be made available within 3 months from initial request to researchers who provide a methodologically sound proposal. The data will be provided after their de-identification, in compliance with applicable privacy laws, data protection, and requirements for consent and anonymization. Transcripts from individual participant interviews will not be made available as patients did not provide consent for these to be shared. Requests for data should be sent to the corresponding author.

## Abbreviations

AE-QoL: Angioedema Quality of Life Questionnaire
ANOVA: analysis of variance
EQ-5D: EuroQol-5 Dimension
FDA: Food and Drug Administration
HAE: hereditary angioedema
HAEA-QoL: United States Hereditary Angioedema Association Quality of Life Questionnaire
HAE PRO: Hereditary Angioedema Patient-Reported Outcomes Questionnaire
HAE-QoL: Hereditary Angioedema Quality of Life Questionnaire
HRQoL: health-related quality of life
ICC: intraclass coefficient
MDC: minimal detectable change
PRO: patient-reported outcome
SD: standard deviation
SDS: Sheehan Disability Scale
SEM: standard error of the mean
VAS: visual analog scale

## Notes

The original version of this article was revised: “In the author group Joan Mendivil was wrongly written as Menauthoril. This has been corrected.”
