# Correction to: Content validation and psychometric evaluation of the Angioedema Quality of Life Questionnaire for hereditary angioedema

**DOI:** 10.1186/s41687-023-00587-7

**Published:** 2023-06-05

**Authors:** Magdalena Vanya, Maureen Watt, Saeid Shahraz, Charlotte E. Kosmas, Stephanie Rhoten, Sara Costa-Cabral, Joan Mendivil, Giovanna Devercelli, Karsten Weller

**Affiliations:** 1 ICON plc, South San Francisco, CA USA; 2 Takeda Pharmaceuticals International AG, Zurich, Switzerland; 3 Tufts Medical Center Institute for Clinical Research and Health Policy, Boston, MA USA; 4 ICON plc, Reading, UK; 5 IQVIA, San Francisco, CA USA; 6 Mapi Research Trust, Lyon, France; 7 Takeda Development Center Americas, Inc, Lexington, MA USA; 8 Institute of Allergology, corporate member of Freie, Charité – Universitätsmedizin Berlin, Universität Berlin and Humboldt-Universität zu Berlin, Berlin, Germany; 9 Fraunhofer Institute for Translational Medicine and Pharmacology ITMP, Immunology and Allergology, Berlin, Germany

**Correction to**: ***J Patient Rep Outcomes*7, 33 (2023).**

10.1186/s41687-023-00576-w

Following publication of the original article [1], the authors identified an error in the author name of Joan Mendivil.

The incorrect author’s name is: Joan Menauthoril.

The correct author’s name is: Joan Mendivil.

The author group has been updated above and the original article [1] has been corrected.
