# Supplementary material for: Content validation and psychometric evaluation of the Angioedema Quality of Life Questionnaire for hereditary angioedema
Source: J Patient Rep Outcomes. 2023 Apr 3;7:33. doi: 10.1186/s41687-023-00576-w (PMC10070575; doi:10.1186/s41687-023-00576-w)
Supplement: Supplementary file 1 — Additional file 1: Supplemental Tables S1–S9. Supplemental Figures S1–S3. [file 41687_2023_576_MOESM1_ESM.docx]

**Additional File 1**

**Supplemental Method S1** Inclusion and exclusion criteria

Eligible patients met the following study inclusion criteria:

1. Confirmed diagnosis of HAE (confirmation using a photographed/photocopy of patient’s prophylactic or rescue medication or prescription for HAE)
2. Experienced an HAE attack in the last 12 months
3. Male or female, aged 9 years or older at screening (note: the analysis presented here includes only patients aged 18 years or older)
4. Willing and able to provide informed verbal consent/assent
5. Able to read and speak the country’s native language fluently
6. Willing to be audio-recorded
7. Willing and able to complete an interview of approximately up to 90 minutes’ duration, either over the phone or in-person
8. The patient, and/or patient’s parent(s) or legally authorized guardian(s) voluntarily provided verbal informed consent/assent, as applicable, after all relevant aspects of the study had been explained and discussed with the patient and/or the patient’s parent(s) or legally authorized guardian(s)
9. The patient, and/or patient’s parent(s) or legally authorized guardian(s) was able to understand the nature, scope, and possible consequences of the study, and agreed to comply with the interview

Patients were ineligible for the study if they met any of the following criteria:

1. Patients with Type 3 HAE (HAE with normal C1-INH)
2. Any medical or psychiatric illness, or indication of cognitive impairment, that could, in the opinion of the study staff, potentially interfere with the patient’s ability to participate in an interview

*C1-INH* C1 inhibitor, *HAE* hereditary angioedema.

**Supplemental Table S1** PRO instruments for HRQoL in HAE

| **Instrument** | **Description** |
| --- | --- |
| Angioedema Quality of Life Questionnaire (AE-QoL) [1-5] | - Evaluates impairment of QoL in patients with recurrent angioedema (HAE and other forms of recurrent AE) - Contains 17 items in 4 domains (functioning, fatigue/mood, fears/shame, food) - Scoring from 0 to 100, with higher scores indicating greater impairment. - 4-week recall period - Sensitivity to change and minimal clinically important difference established - Validation limited to patients in Germany, Thailand, Japan, and Turkey |
| Health-related Quality of Life Questionnaire for Hereditary Angioedema (HAE-QoL) [6, 7] | - Developed for cross-cultural use to assess HRQoL in patients with C1-INH-HAE - Development included patients from 18 countries (Argentina, Austria, Brazil, Canada, China, Denmark, France, Germany, Hungary, Israel, Italy, Macedonia, Netherlands, Panama, Poland, Romania, Spain, United Kingdom) - Contains 25 items in seven dimensions (treatment difficulties, physical functioning and health, disease related stigma, emotional role and social functioning, concern about offspring, perceived control over illness, and mental health) - Total score ranges from 25–135 (range of scores differs between the various dimensions); higher scores indicate lower impairment. - 6-month recall period |
| HAE Patient Reported Outcomes Questionnaire (HAE PRO) [8] | - Developed to assess patients’ experience with HAE attacks, including symptoms, treatment, use of healthcare resources, and impact on patients’ lives. - Development included patients from six countries (Argentina, Brazil, France, Germany, United Kingdom, United States) - 18 items in seven concepts (HAE attack, attack triggers, attack warning signs, resource use, attack location, attack impact, attack symptoms) - Patients complete the questionnaire ideally within days following an attack |
| United States HAE Association (HAEA)-QoL (HAEA-QoL) [9] | - Developed to evaluate HRQoL in patients in the United States - Contains 27 items in 2 domains (feelings, concerns) - Higher scores indicate poorer outcome |

*HAE* hereditary angioedema, *HRQoL* health-related quality of life, *PRO* patient-reported outcome

**REFERENCES**

1. Weller K, Groffik A, Magerl M et al. (2012) Development and construct validation of the angioedema quality of life questionnaire. Allergy 67:1289-1298. doi:10.1111/all.12007

2. Weller K, Magerl M, Peveling-Oberhag A, Martus P, Staubach P, Maurer M (2016) The Angioedema Quality of Life Questionnaire (AE-QoL) – assessment of sensitivity to change and minimal clinically important difference. Allergy 71:1203-1209. doi:10.1111/all.12900

3. Kulthanan K, Chularojanamontri L, Rujitharanawong C, Weerasubpong P, Maurer M, Weller K (2019) Angioedema quality of life questionnaire (AE-QoL) - interpretability and sensitivity to change. Health Qual Life Outcomes 17:160. doi:10.1186/s12955-019-1229-3

4. Morioke S, Takahagi S, Kawano R et al. (2021) A validation study of the Japanese version of the Angioedema Activity Score (AAS) and the Angioedema Quality of Life Questionnaire (AE-QoL). Allergol Int 70:471-479. doi:10.1016/j.alit.2021.04.006

5. Can PK, Degirmentepe EN, Etikan P et al. (2021) Assessment of disease activity and quality of life in patients with recurrent bradykinin-mediated versus mast cell-mediated angioedema. World Allergy Organ J 14:100554. doi:10.1016/j.waojou.2021.100554

6. Prior N, Remor E, Gómez-Traseira C et al. (2012) Development of a disease-specific quality of life questionnaire for adult patients with hereditary angioedema due to C1 inhibitor deficiency (HAE-QoL): Spanish multi-centre research project. Health Qual Life Outcomes 10:82. doi:10.1186/1477-7525-10-82

7. Prior N, Remor E, Pérez-Fernández E et al. (2016) Psychometric Field Study of Hereditary Angioedema Quality of Life Questionnaire for Adults: HAE-QoL. J Allergy Clin Immunol Pract 4:464-473.e4. doi:10.1016/j.jaip.2015.12.010

8. Bonner N, Abetz-Webb L, Renault L et al. (2015) Development and content validity testing of a patient-reported outcomes questionnaire for the assessment of hereditary angioedema in observational studies. Health Qual Life Outcomes 13:92. doi:10.1186/s12955-015-0292-7

9. Busse PJ, Christiansen SC, Birmingham JM et al. (2019) Development of a health-related quality of life instrument for patients with hereditary angioedema living in the United States. J Allergy Clin Immunol Pract 7:1679-1683.e7. doi:10.1016/j.jaip.2018.11.042

**Supplemental Table S2** Hereditary angioedema-related patient impacts extracted from the literature

| **Domain** | **Concepts** | **Frequency *N*=28**  ***n* (%)** |
| --- | --- | --- |
| Daily activities | Impaired ability to perform or complete daily activities | 4 (14.3) |
|  | Difficulty performing household tasks | 3 (10.7) |
|  | Difficulty eating | 2 (7.1) |
|  | Difficulty driving | 2 (7.1) |
|  | Difficulty leaving house | 2 (7.1) |
|  | Difficulty typing due to swelling | 1 (3.6) |
|  | Difficulty wearing clothes/shoes/jewelry | 1 (3.6) |
|  | Difficulty using a computer | 1 (3.6) |
|  | Difficulty with self-care | 1 (3.6) |
| Work/school | Missing work/school due to attack (absenteeism) | 10 (35.7) |
| Emotional | Fear of asphyxiation | 2 (7.1) |
|  | Embarrassment | 2 (7.1) |
| Financial | Cost of treatment | 2 (7.1) |
|  | Loss of earnings due to work impacts | 2 (7.1) |
|  | Cost of hospitalizations | 1 (3.6) |
| Social | Miss social events due to attacks | 1 (3.6) |
|  | Reduced social function | 1 (3.6) |
|  | Stigma of swollen face | 1 (3.6) |
|  | Lack of understanding from others | 1 (3.6) |
| Physical | Reduced mobility (difficulty standing/walking, limited use of feet) | 3 (10.7) |
|  | Tiredness/exhaustion | 2 (7.1) |
|  | Reduced dexterity (limited use of hands) | 2 (7.1) |
|  | Incapacitated/bedridden | 2 (7.1) |
|  | Reduced physical function | 2 (7.1) |
|  | Difficulty sitting | 1 (3.6) |
|  | Difficulty speaking | 1 (3.6) |
| Cognitive | Difficulty with concentration | 2 (7.1) |
| Role | Dependent on others | 2 (7.1) |
|  | Difficulty caring for children | 1 (3.6) |
|  | Inability to participate in family activities | 1 (3.6) |
| Leisure | Difficulty participating in leisure activities | 2 (7.1) |

**Supplemental Table S3**. Hereditary angioedema-related impacts in between attacks extracted from the literature

| Domain | Concepts | Frequency N=28  *n* (%) |
| --- | --- | --- |
| Daily living | Reluctance to travel due to fear of attack | 4 (14.3) |
| Work/School | Hindered advancement | 6 (21.4) |
|  | Impaired productivity | 5 (17.9) |
|  | Inability to work full-time | 1 (3.6) |
|  | Inability to perform job tasks | 1 (3.6) |
|  | Discrimination/lack of understanding from co-workers | 1 (3.6) |
|  | Could not consider certain jobs due to HAE | 1 (3.6) |
|  | Unable to keep job | 1 (3.6) |
|  | Unable to get job | 1 (3.6) |
|  | Takes longer to complete education | 1 (3.6) |
| Emotional | Depression | 6 (21.4) |
|  | Fear/worrying^a^ | 5 (17.9) |
|  | Anxiety | 5 (17.9) |
|  | Self-image | 2 (7.1) |
|  | Frustration | 1 (3.6) |
|  | Mood variability | 1 (3.6) |
|  | Anger | 1 (3.6) |
| Financial | Cost of hospital appointments | 3 (10.7) |
|  | Cost of travel for treatment | 2 (7.1) |
|  | Cost of medications | 1 (3.6) |
|  | Cost of injection supplies | 1 (3.6) |
| Social/leisure | Avoidance of social opportunities | 3 (10.7) |
|  | Need to avoid certain hobbies | 1 (3.6) |
|  | Others don’t understand HAE | 2 (7.1) |
|  | Inability to plan ahead | 1 (3.6) |
|  | Social impairment | 1 (3.6) |
| Physical | Irregular menstruation | 1 (3.6) |
| Relationships | Strain on family relationships | 1 (3.6) |
|  | Strain on intimate relationships | 1 (3.6) |
|  | Relationship impairment | 1 (3.6) |
| Role | Have no or fewer than desired children | 1 (3.6) |
| Quality of life | Reduced quality of life | 2 (7.1) |
|  | Lack of known trigger which can be avoided | 1 (3.6) |

^a^ For example, of passing disease to children, of future attacks, about treatment side effects

**Supplemental Table S4**. Hereditary angioedema-related treatment-related impacts extracted from the literature

| Domain | Concepts | Frequency N*=*28 *n* (%) |
| --- | --- | --- |
| Treatment | Need for hospitalization | 8 (28.6) |
|  | Side effects^a^ | 5 (17.9) |
|  | Unnecessary treatments or surgical procedures due to misdiagnosis as appendicitis, arthritis, food allergies, stomach ulcers | 5 (17.9) |
|  | Need for emergency department visits | 5 (17.9) |
|  | Dissatisfaction with management of disease | 2 (7.1) |
|  | Need to travel to hospital/clinic for preventative treatment | 2 (7.1) |
|  | Health care providers do not know about condition | 2 (7.1) |
|  | Need to use health care provider in between attacks | 1 (3.6) |
|  | Interference with taking other treatments (e.g., oral contraception) | 1 (3.6) |
|  | Unnecessary psychiatric referrals due to misdiagnosis as psychosomatic | 1 (3.6) |
|  | Misdiagnosis due to rare condition | 1 (3.6) |
|  | Experimental treatment due to rare condition | 1 (3.6) |
|  | Reluctance of ED personnel to administer C1-INH due to lack of familiarity with drug | 1 (3.6) |
|  | Difficulty of ED obtaining C1-INH due to being rare drug | 1 (3.6) |
|  | Decreased interest in ED personnel due to repeated patient visits | 1 (3.6) |

*C1-INH* C1 inhibitor, *ED* emergency department

^a^ Headaches, diarrhea, pyrexia, nasal congestion, anaphylaxis, depression, hypertension, liver adenomas, menstrual irregularities, weight gain, virilization, pruritis, rash, light-headedness, fever, mood changes, agitation, insomnia, increased hair growth, acne, sexual side effects, increase in cholesterol levels, changes in attention span, liver function problems, voice changes, blood clots, stunted growth

**Supplemental Table S5**. Summary of patient characteristics (patient interviews)

| Characteristic | *N* (%) |
| --- | --- |
| Country |  |
| United Kingdom | 11 (27.50) |
| United States | 12 (30.0) |
| Canada | 4 (10.0) |
| Germany | 5 (12.5) |
| France | 4 (10.0) |
| Spain | 4 (10.0) |
| Gender |  |
| Female | 35 (87.5) |
| Male | 5 (12.5) |
| Age, years |  |
| Mean (SD) | 39.25 (13.43) |
| Range | 18–66 |
| Race^a^ |  |
| White | 28 (90.3) |
| Asian | 2 (6.5) |
| Other | 1 (3.2) |
| Number of attacks/year | N=25 |
| 1–5 | 3 (12) |
| 6–15 | 8 (32) |
| 19–40 | 7 (28) |
| 100 or more | 3 (12) |
| “Once every 2 months” | 1 (4) |
| “Average 2 to 3 a month” | 1 (4) |
| “Every 2-3 days” | 1 (4) |
| “Countless” | 1 (4) |

^a^ Information collected only in UK, US, Canada, and Spain (n=31).

Country-specific categories provided relevant to their country of residence.

**Supplemental Table S6** Summary of attack symptoms reported by patients during interviews

| **Symptom, *n* (%)** | **Patients (*N*=40)** |
| --- | --- |
| Vomiting/nausea | 30 (75.0) |
| Pain | 27 (67.5) |
| Swelling | 27 (67.5) |
| Difficulty breathing/blocked airway | 12 (30.0) |
| Diarrhea | 11 (27.5) |
| Skin tightness | 10 (25.0) |
| Tiredness | 10 (25.0) |
| Cramping | 8 (20.0) |
| Rash | 6 (15.0) |
| Itching | 4 (10.0) |
| Difficulty swallowing | 4 (10.0) |
| Numbness/tingling | 4 (10.0) |
| Difficulty talking | 3 (7.5) |
| Bowel wall obstruction | 3 (7.5) |
| Fainting/passing out | 3 (7.5) |
| Bloating | 3 (7.5) |
| Hot sensation | 3 (7.5) |
| Vision problems | 3 (7.5) |
| Dizziness | 2 (5.0) |
| Heavy feeling | 2 (5.0) |
| Headache | 2 (5.0) |
| Skin redness | 2 (5.0) |
| Skin sensitivity | 2 (5.0) |
| Dehydration | 2 (5.0) |
| Bruising/tenderness | 2 (5.0) |
| Voice change | 2 (5.0) |
| Stiffness | 2 (5.0) |
| Other symptoms^a^ | 1 (2.5) |

^a^ Symptoms each reported by only one patient included mood change, sensitivity to environment, circulation problems, generally feeling unwell, blood in stools, constipation, an acidic/burning feeling, cold fingertips, hiccups, prolapsed colon

**Supplemental Table S7** Summary of item total correlations for AE-QoL total and domain scores at baseline

| **AE-QoL Item [n=64]** | **Spearman Rank Correlation** | | | | |
| --- | --- | --- | --- | --- | --- |
|  | **Total score** | **Functioning** | **Fatigue/Mood** | **Fears/Shame** | **Food** |
| Item 1 (Impairment of work) | 0.63*** | 0.76*** |  |  |  |
| Item 2 (Impairment of physical activity) | 0.63*** | 0.85*** |  |  |  |
| Item 3 (Impairment of spare time activities) | 0.72*** | 0.89*** |  |  |  |
| Item 4 (Impairment of social relations) | 0.65*** | 0.84*** |  |  |  |
| Item 5 (General limitations in foods and eating) | 0.68*** |  |  |  | 0.89*** |
| Item 6 (Difficulties of falling asleep) | 0.57*** |  | 0.76*** |  |  |
| Item 7 (Waking up during the night) | 0.64*** |  | 0.80*** |  |  |
| Item 8 (Feeling tired during the day) | 0.67*** |  | 0.83*** |  |  |
| Item 9 (Difficulties in concentrating) | 0.60*** |  | 0.72*** |  |  |
| Item 10 (Feeling depressed) | 0.64* |  | 0.68*** |  |  |
| Item 11 (Limitations in the selection of food and beverages) | 0.65*** |  |  |  | 0.92*** |
| Item 12 (Feeling burdened at having swellings) | 0.75*** |  |  | 0.80*** |  |
| Item 13 (Fear of new suddenly appearing swellings) | 0.73*** |  |  | 0.83*** |  |
| Item 14 (Fear of increased frequency of swellings) | 0.74*** |  |  | 0.85*** |  |
| Item 15 (Ashamed to visit public places) | 0.63*** |  |  | 0.80*** |  |
| Item 16 (Embarrassed by the appearance of swellings) | 0.65*** |  |  | 0.79*** |  |
| Item 17 (Fear of long-term negative drug effects) | 0.52*** |  |  | 0.56*** |  |

**p*<0.05; ****p*<0.001

**Supplemental Table S8**. Correlations between change in SDS total scores with change in AE-QoL total and domain scores

| **SDS Total Score Change** | ***N*** | **AE-QoL Total Score** | **AE-QoL Functioning** | **AE-QoL Fatigue/Mood** | **AE-QoL Fears/Shame** | **AE-QoL Food** |
| --- | --- | --- | --- | --- | --- | --- |
| Baseline to Week 13^a^ | 40 | 0.40* | 0.39*** | 0.23 | 0.30 | 0.37* |
| Week 5 to Week 13^b^ | 36 | 0.51** | 0.38* | 0.42* | 0.42** | 0.35* |

*AE-QoL* Angioedema Quality of Life Questionnaire, *SDS* Sheehan Disability Scale

**p*<0.05, ***p*<0.01, ****p*<0.001

^a^ Change in scores calculated as Week 13 − Baseline

^b^ Change in scores calculated as Week 13 − Week 5

**Supplemental Table S9** Distribution-based estimates of meaningful change for the AE-QoL at Week 5

| **AE-QoL Score** | **Week 5** | **0.5 SD** | **SEM: Alpha** | **SEM: Test-Retest** | **MDC: Alpha** | **MDC: Test-Retest** |
| --- | --- | --- | --- | --- | --- | --- |
| Total | 23.74 | 11.87 | 5.35 | 7.87 | 12.49 | 18.70 |
| Functioning | 27.03 | 13.51 | 7.06 | 11.14 | 16.46 | 26.48 |
| Fatigue/mood | 25.96 | 12.98 | 8.70 | 11.31 | 20.31 | 26.88 |
| Fears/shame | 27.55 | 13.77 | 8.65 | 9.54 | 20.19 | 22.67 |
| Food | 30.80 | 15.40 | 13.68 | 10.67 | 31.93 | 25.35 |

*AE-QoL* Angioedema Quality of Life Questionnaire, *MDC* minimum detectable change, *SD* standard deviation, *SEM* standard error of the mean

Notes: SEM calculated as 0.5 SD × sqrt(1−reliablity); MDC calculated as 1.96 × SEM × sqrt(2)

**Supplemental Figure S1** Literature review (2016) flow chart


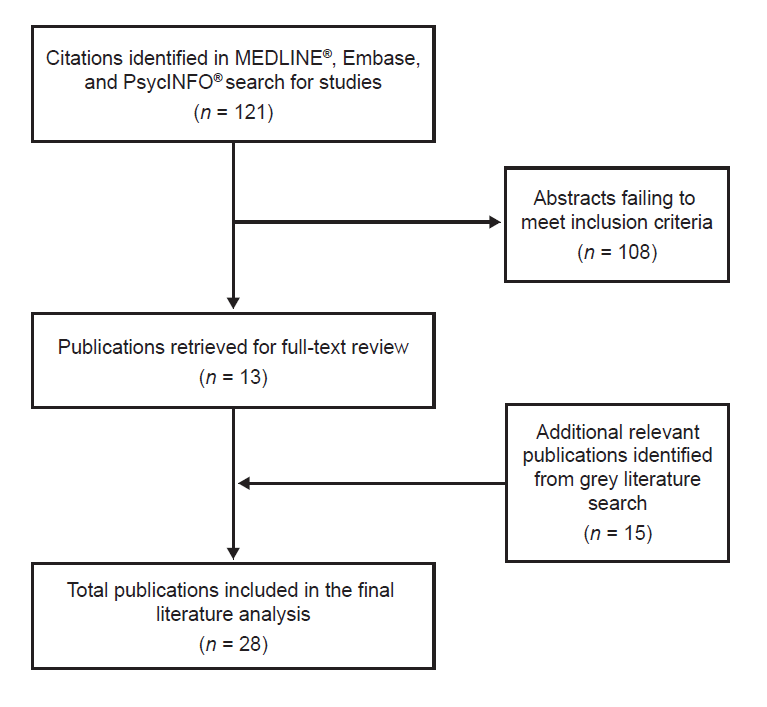


The MEDLINE^®^, Embase, and PsycINFO^®^ databases were searched on July 20, 2016. Google Scholar, conference proceedings, and article reference lists were also searched. A total of 121 abstracts were identified as potentially relevant, of which 13 were selected for full-text review based on inclusion/exclusion criteria and internal discussion. Following the initial round of full-text publication review, four additional publications were identified from the reference lists of publications already included in the analysis, eight publications were identified through Google Scholar searches, and three publications were identified from a review of relevant conference proceedings. This resulted in a total of 28 publications reviewed in full. Of these, 20 were peer-reviewed journal articles, six were conference abstracts, and two were conference presentations. Seven of the 28 publications were qualitative studies while the remaining 21 were non-qualitative studies such as observational or survey studies.

**Supplemental Figure S2** Literature review (2020) flow chart


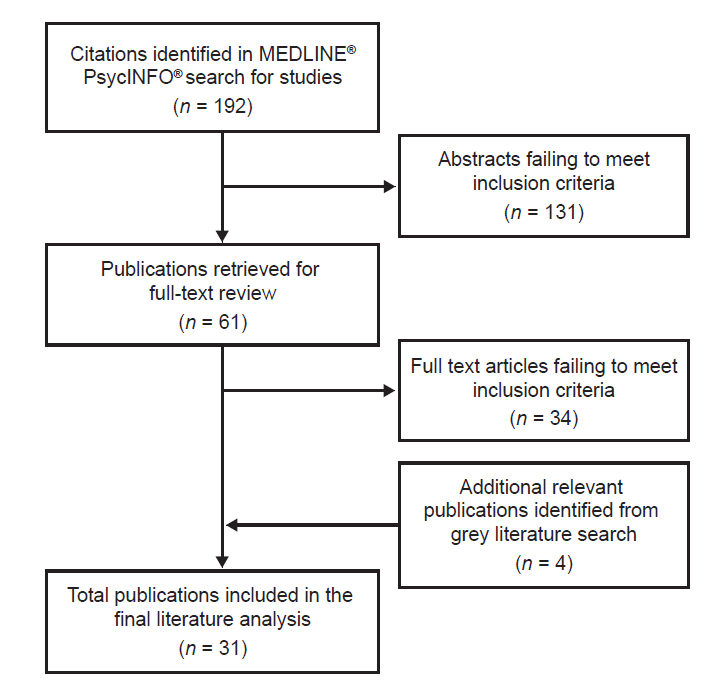


The search was performed on April 27, 2020 in the MEDLINE^®^ and PsycInfo^®^ databases. A total of 192 retrieved references were reviewed and selected, and 31 references were selected for data extraction.

**Figure S3**. AE-QoL score distributions at baseline for the psychometric validation (n=64)


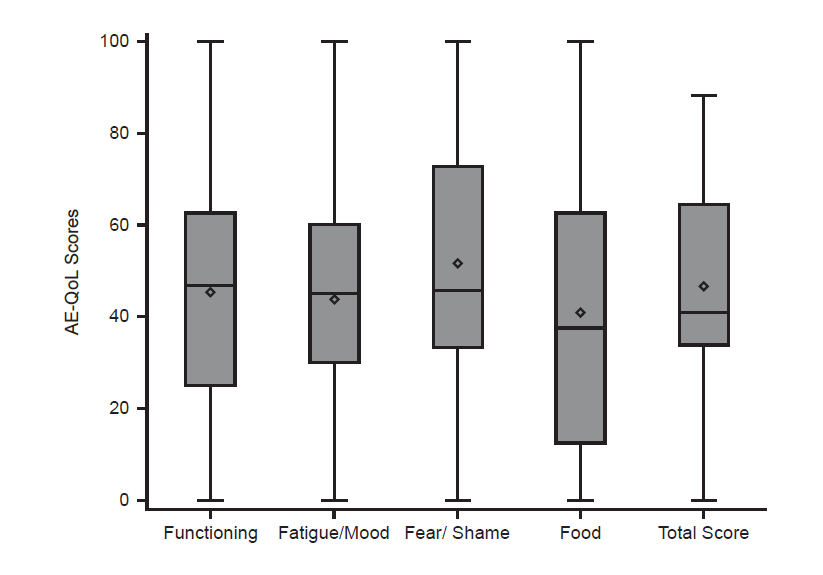


Whiskers and boxes represent the minimum, 25^th^ percentile, median, 75^th^ percentile, and maximum. The diamond represents the mean.
